# Human PMNs exhibit greater engulfment, NETosis, and enhanced migration when incubated with nontypeable *Haemophilus influenzae* newly released from a biofilm

**DOI:** 10.3389/fmicb.2025.1728903

**Published:** 2025-11-27

**Authors:** Kathryn Q. Wilbanks, Joshua Flesher, Steven D. Goodman, Lauren O. Bakaletz

**Affiliations:** 1 Center for Microbe and Immunity Research, Abigail Wexner Research Institute at Nationwide Children’s Hospital, Columbus, OH, United States; 2 Department of Pediatrics, The Ohio State University College of Medicine, Columbus, OH, United States

**Keywords:** DNABII, NRel, freshly dispersed, LOS, DNA, IL-8, timelapse microscopy, flow cytometry

## Abstract

Biofilm-resident bacteria exhibit diverse mechanisms to evade eradication, including the highly protective self-produced matrix in which they are embedded. Thus, releasing bacteria from biofilm residence affords antibiotics and immune effectors greater access. We developed a monoclonal antibody directed against an essential biofilm matrix protein that induces rapid collapse of the biofilm matrix with release of bacteria that are in a transient but highly vulnerable phenotype. Bacteria that are newly released (“NRel”) due to this monoclonal are significantly more sensitive to antibiotic-, antimicrobial peptide- or human PMN-mediated killing *in vitro* and are rapidly eradicated in four animal models without adjunct antibiotic treatment, the speed of which highlights the role of innate immune effectors. Here, we characterized the reactivity of human PMNs to three distinct bacterial populations derived from three clinical isolates of the predominant respiratory tract pathogen nontypeable *Haemophilus influenzae* (NTHI). Via timelapse microscopy and flow cytometry, PMN engulfment, NETosis (e.g., programmed neutrophil cell death accompanied by release of web-like condensed DNA with associated antimicrobial proteins), and migratory activity were assessed when PMNs were incubated with NTHI that were dual-fluorescently labeled with green-fluorescent FM 1–43 and pH-sensitive red-fluorescent pHrodo™ Red, SE, which fluoresces in acidic environments such as within a phagolysosome. Relative concentrations of endotoxin and DNA, as well as cytokines/chemokines potentially responsible for observed PMN activities were also assessed. PMN-mediated engulfment, NETosis, and migratory activity were all greatest when incubated with NTHI NRel compared to both NTHI that had been grown planktonically in rich medium or to NTHI that were present in the culture medium that overlayed the biofilm. Whereas neither NTHI endotoxin nor DNA played a role in the observed relative activities, PMNs incubated with NTHI NRel released significantly more IL-8 which likely served to enhance the migration of additional PMNs. These data contribute to our understanding of mechanisms anticipated to be operational in the DNABII protein-targeted monoclonal antibody-based treatment regimen we developed to facilitate host-mediated contribution to biofilm eradication after induced release of formerly biofilm-resident bacteria into the highly vulnerable NRel state.

## Introduction

Bacterial biofilms are implicated in a substantial proportion of bacterial infections, particularly those that are chronic, recurrent, recalcitrant to treatment or device-related (Di Domenico et al., 2022). Importantly, biofilm-resident bacteria are significantly more tolerant to antibiotics and effectors of the host immune system (Mirghani et al., 2022; Yang et al., 2023). In fact, bacteria within biofilms are 10 to 1,000-fold less susceptible to antimicrobial agents than their isogenic planktonically growing counterparts, which often ultimately leads to ineffective medical management, prolonged disease course, delays in time to healing, or even the need for invasive interventions to attempt infection resolution (Uruén et al., 2020).

To more effectively resolve biofilm-related diseases, development of novel approaches are of great priority and urgency and many laboratories are working toward such goals (Shrestha et al., 2022). In our lab, we’ve been focused on the ubiquitous DNABII family of bacterial DNA-binding and -bending proteins that function as architectural elements both inside and outside the bacterial cell. The two DNABII proteins, histone-like protein (HU) and integration host factor (IHF), lend essential structural support to the lattice-like network of extracellular DNA (eDNA) in the biofilm matrix as they are positioned at the vertices of crossed strands of eDNA and thereby serve as linchpin proteins that bind to, bend, and stabilize double-stranded eDNA (Swinger and Rice, 2004; Dey et al., 2017; Devaraj et al., 2018). As DNABII proteins are essential components of the biofilm matrix, we developed a monoclonal antibody (mAb) directed against a synthetic “tip-chimer” peptide immunogen designed to mimic the immunoprotective DNA-binding “tips” of a DNABII protein as determined by epitope mapping studies (Goodman et al., 2011; Novotny et al., 2016, 2019). When biofilms formed *in vitro* by a diverse array of human pathogens are incubated with this mAb (“α-DNABII”), an equilibrium shift occurs wherein free DNABII proteins that have come off of the biofilm matrix are then bound by the mAb which prevents their ability to rebind to the eDNA within the biofilm matrix. As a result, the structural lattice is destabilized which leads to significant biofilm collapse with concomitant rapid release of formerly biofilm-resident bacteria (Jurcisek et al., 2022; Kurbatfinski et al., 2022, Kurbatfinski et al., 2023a; Rhodes et al., 2024).

Regardless of the method or agent used, many laboratories report that bacteria which have recently exited or been forced out of biofilm residence exhibit a transient, yet distinct, phenotype that often includes significantly increased susceptibility to antibiotic-mediated killing relative to planktonically grown counterparts (Marks et al., 2013; Li et al., 2014; Petrova and Sauer, 2016; Chambers et al., 2017; Goodwine et al., 2019; Rumbaugh and Sauer, 2020; Zemke et al., 2020; Kalia and Sauer, 2024). This enhanced sensitivity characteristic is of great potential clinical relevance given that, historically, planktonically grown bacteria have been considered to be the most antibiotic sensitive. Unfortunately it is the planktonic bacterial lifestyle that is relied upon to determine treatment protocols that too often fail when attempting to eradicate biofilm-resident bacteria. After α-DNABII-induced biofilm disruption, we too have characterized a phenotype of significantly increased antibiotic susceptibility in bacteria newly released from biofilms formed by any of 24 diverse genera of pathogens, including all members of the high priority and multiple-antibiotic-resistant ESKAPEE pathogens (Mokrzan et al., 2018, 2020; Kurbatfinski et al., 2022, Kurbatfinski et al., 2023a,b; Wilbanks et al., 2023). While we have not yet fully elucidated all of the molecular mechanisms that underlie the marked sensitivity of α-DNABII NTHI NRel to antibiotic killing, we have previously reported that they appear to be in canonical lag phase of growth and exhibit both significantly increased outer membrane permeability and significantly downregulated expression of at least two genes involved in mediation of oxidative stress compared to planktonic NTHI, among other findings (Wilbanks et al., 2023).

In addition to its *in vitro* disruptive capability, we have shown in four distinct pre-clinical models of human disease that α-DNABII treatment facilitates significant disease-resolution efficacy. Therapeutic treatment with α-DNABII resolves mucosal biofilms formed by *Aggregatibacter actinomycetemcomitans* in a rat osteolytic peri-implantitis model (Freire et al., 2017), facilitates clearance of aggregate *Pseudomonas aeruginosa* and *Mycobacterium abscessus* biofilms from the murine lung (Jurcisek et al., 2025, Novotny et al., 2016), and antigen-binding fragments derived from a humanized α-DNABII mAb mediate resolution of nontypeable *Haemophilus influenzae* (NTHI)-induced otitis media in the chinchilla middle ear (Novotny et al., 2021). Preventatively, active immunization of chinchillas with the tip-chimer peptide itself induces formation of antibodies that effectively disrupt existing NTHI biofilms in the middle ear, which ultimately leads to rapid resolution of experimental otitis media (Novotny et al., 2019).

Intriguingly, after therapeutic treatment with α-DNABII, the rapid biofilm eradication and disease resolution we observed in each of these pre-clinical models occurred without use of added antibiotics. This common outcome suggested to us that the primary means of infection resolution was likely due to the action of innate immune effectors. As such, we wondered whether NTHI newly released from biofilm residence by α-DNABII (i.e., “α-DNABII NTHI NRel”) might, in addition to being significantly more susceptible to both antibiotic- and antimicrobial peptide-mediated killing, also display a phenotype of increased susceptibility to human PMNs, which arrive rapidly at infection sites (Liew and Kubes, 2019). Whereas both planktonic and biofilm resident NTHI have been shown to resist killing by PMNs (Hong et al., 2009; Juneau et al., 2011), which includes the ability of biofilm-resident NTHI to utilize DNABII proteins in an offensive manner to convert native NET B-form DNA to the rare Z-form and thereby abrogate NET killing function (Buzzo et al., 2021), we’ve shown that NRel NTHI are significantly more susceptible to killing by human PMNs in an NADPH-oxidase sensitive manner (Wilbanks et al., 2023), which is likely related to the fact that α-DNABII NTHI NRel exhibit significantly downregulated expression of gene products involved in their ability to mediate oxidative stress (Wilbanks et al., 2023).

This is not surprising given that there have been multiple reports which characterize the highly unique phenotype of bacterial cells that have newly dispersed from biofilm residence (Rollet et al., 2009; Chua et al., 2014; Barraud et al., 2015; Kalia and Sauer, 2024). As such, we were interested in how human PMNs responded when incubated with either planktonically grown NTHI, those NTHI newly released from biofilm residence by anti-DNABII or NTHI that were present in the medium that overlaid a biofilm. The latter population was hypothesized to be more similar to those bacteria found in disease sites such as a middle ear effusion or within synovial fluid of an infected joint than those grown planktonically and heretofore will be referred to as non-biofilm associated (“NBfA”) NTHI. Herein, via timelapse microscopy and flow cytometry analysis, we qualified and quantified, respectively, human PMN uptake of NTHI, NETosis, and migratory activity when incubated with each of these three NTHI populations. We also monitored for relative concentrations of both NTHI endotoxin and DNA within the fluids in which these populations were suspended when incubated with PMNs as both are known to induce NETosis (Juneau et al., 2011). Further, we assayed for the relative release of chemokines/cytokines by PMNs when incubated with each of the three tested NTHI populations.

## Materials and methods

### Ethics

De-identified human blood donations provided by healthy adult subjects that span the demographic spectrum of central Ohio were made under the auspices of the Research Institute Blood Donor Services of Nationwide Children’s Hospital after informed written consent was obtained. PMNs were isolated from these blood specimens for use in studies conducted within our laboratory in conformity with, and as approved under, Nationwide Children’s Hospital Institutional Biosafety Committee (IBC) protocol #IBS-00000449. All experiments were conducted in accordance with relevant guidelines and regulations of the Nationwide Children’s Hospital Research Institute Blood Donor Services and Institutional Biosafety Committee.

### Bacterial strains and growth

Nontypeable *Haemophilus influenzae* (NTHI) strain 86-028NP (Sirakova et al., 1994) was maintained frozen in LN_2_ at passage #4 on artificial medium since its original isolation from the nasopharynx of a child who underwent tympanostomy tube insertion due to chronic otitis media. For fluorescent timelapse microscopy and flow cytometry, GFP-expressing NTHI 86-028NP/pRSM2211 (Mason et al., 2003) was utilized. NTHI strains 1128 and 1728, also isolated from children with chronic otitis media (Bakaletz et al., 1988) had been maintained in LN_2_ at passage #4 or #7, respectively. All NTHI strains were grown in Brain Heart Infusion (Thermo Fisher Scientific, Waltham, MA) broth supplemented with hemin (2 μg/mL) (Sigma-Aldrich, St. Louis, MO) and β-NAD (2 μg/mL) (Sigma-Aldrich) (e.g., sBHI) or on chocolate agar (Thermo Fisher Scientific) at 37 °C with 5% CO_2_ in a humidified atmosphere.

### PMN isolation and preparation

Human neutrophils were isolated from whole blood via magnetic negative selection using the EasySep™ Direct Human Neutrophil Isolation Kit (StemCell Technologies, Inc., Vancouver, Canada). The final PMN pellet was resuspended in 1 mL HBSS (Thermo Fisher Scientific) and total number PMNs/mL HBSS enumerated before making separate solutions of 1e6 PMNs/mL HBSS for timelapse microscopy or 2e6 PMNs/mL HBSS for flow cytometry.

### Antibodies

A murine monoclonal antibody of the IgG isotype against a 44-mer tip-chimer peptide designed to mimic protective epitopes of the DNA-binding “tips” of the alpha and beta subunits of a bacterial DNABII protein were prepared for us under contract to Rockland Immunochemicals, Inc., (Philadelphia, PA) and has been previously characterized (Novotny et al., 2016). This monoclonal antibody is referred herein as “MsTipMab.”

### Collection of α-DNABII NRel, NBfA, or planktonic NTHI

For NTHI 86-028NP/pRSM2211 or 1728, biofilms were established as previously described (Wilbanks et al., 2023) after seeding 10 cm^2^ flat tissue culture tubes (TPP, Trasadingen, Switzerland) with 2e5 CFU/mL solution for 16 h. As NTHI 1128 exhibited growth approximately two times that of either NTHI 86-028NP/pRSM2211 or 1728 (as evidenced by growth curve analysis), NTHI 1128 was seeded as previously described (Wilbanks et al., 2023) but at 1e5 CFU/mL. Regardless of NTHI strain, after 16 h biofilms were gently washed to remove non-adherent bacterial cells prior to incubation for 2 h with 5 μg MsTipMab diluted in sBHI/0⋅8 cm^2^ at 37 °C with 5% CO_2_ in a humidified atmosphere for collection of α-DNABII NTHI NRel. Planktonically grown NTHI 86-028NP/pRSM2211 or 1728 were allowed to grow statically in sBHI broth at 37 °C with 5% CO_2_ in a humidified atmosphere for 3 h which was pre-determined to be mid-log phase. NTHI 1128 was allowed to grow statically at 37 °C with 5% CO_2_ in a humidified atmosphere for 1.5 h to mid-log phase due to the faster rate of growth of this isolate. To prepare lag phase planktonically grown bacteria, NTHI 86-028NP/pRSM2211 or 1728 were allowed to grow statically in sBHI at 37 °C with 5% CO_2_ in a humidified atmosphere for 1 h to mid-lag phase. NTHI 1128 was allowed to grow statically in sBHI at 37 °C with 5% CO_2_ in a humidified atmosphere for 30 m to mid-lag phase. Growth phase status was pre-determined for all three NTHI isolates by conducting overnight growth curves. To generate NBfA, NTHI biofilms were established in flat tissue culture tubes for 16 h, gently washed to remove non-adherent bacteria, then incubated for 2 h with 1.25 mL sBHI only before collecting NBfA bacteria present within the fresh media above the biofilm, as previously described (Boisvert et al., 2016; Kragh et al., 2023; Zhao et al., 2023).

### Fluorescent labeling of NTHI

For fluorescent timelapse microscopy and flow cytometry, all NTHI bacterial populations were collected and centrifuged for 3 m at 14,000 rpm at 4 °C. Bacterial pellets were resuspended in 400 μL 0.1M sodium bicarbonate buffer at pH 8.4, centrifuged as before, resuspended in 0.1M sodium bicarbonate buffer, then sonicated for 2 m in a water bath sonicator (Branson Ultrasonics Corporation, Brookfield, CT). All NTHI strains were then labeled with the red fluorescing pH-sensitive dye, pHrodo™ Red, SE (Thermo Fisher Scientific), per manufacturer’s instructions for 30 m at room temperature, protected from light. pHrodo™ Red SE is a pH-sensitive dye that only fluoresces in acidic environments, such as within a phagolysosome where the pH can reach ≤5.0 (Lee et al., 2003), and thus was utilized as an indicator of successful PMN engulfment. After 30 m, for NTHI strains 1128 and 1728 that do not express GFP, these bacterial populations were then also labeled with green fluorescent membrane stain FilmTracer FM™ 1-43 (Invitrogen, Waltham, MA) per manufacturer’s instructions for 15 m at room temperature and protected from light. For strain 86-028NP, we used a constitutive GFP-expressing reporter, (NTHI 86-028NP/pRSM2211), hence referred to as “NTHI 86-028NP” for simplicity (Bakaletz et al., 1988, 1997; Sirakova et al., 1994; DeMaria et al., 1996; Miyamoto and Bakaletz, 1996; Novotny and Bakaletz, 2003). Dual labeling of NTHI strains 1128 and 1728 with FilmTracer FM™ 1-43 was used to ensure that engineering of NTHI 86-028NP/pRSM211 to report GFP was not responsible for the NRel phenotypes observed.

After the labeling periods, all NTHI bacterial populations were centrifuged for 3m at 14,000 rpm at 4 °C. Bacterial pellets were resuspended in 400 μL 0.1M sodium bicarbonate buffer at pH 8.4, centrifuged as before, and the process repeated for a total of two buffer washings. Bacterial pellets were finally resuspended in 0.1M sodium bicarbonate buffer to 1e6 CFU/mL for microscopy or to 4e6 CFU/mL in 0.1M sodium bicarbonate buffer for flow cytometry. Green fluorescent labeling intensity did not differ between NTHI that reported GFP (strain 86-028NP) and those that were labeled with green FilmTracerFM™ (strains 1128 and 1728) during the duration of any of the studies conducted herein, however equivalent photo bleaching would occur for either with prolonged imaging. To ensure similar CFU NTHI/mL were added into each assay, and to ensure bacterial viability, all fluorescently labeled bacterial populations were diluted and plated for CFU determination.

### Timelapse microscopy

Nontypeable *Haemophilus influenzae* (NTHI) bacterial solutions at 1e6 CFU/mL were sonicated for 2m in a water bath sonicator before spotting 10 μL of each bacterial population into separate quadrants of a 4-chambered glass bottom dish (Cellvis, Mountain View, CA). α-DNABII NTHI NRel, NBfA, or planktonically grown NTHI were allowed to adhere to the glass for 30 m at room temperature in a humidified atmosphere before imaging. Immediately before imaging, fluids of the 10 μL droplet were carefully pipetted away, then 10 μL PMN solution at 1e6 PMNs/mL was added. Imaging began as soon as PMNs were added to bacterial spots at 100x magnification on a Nikon Ti2-E White Light inverted microscope (Nikon Instruments Inc., Melville, NY). Images were captured every 30 s until we were able to visualize over-saturation of pHrodo™ red fluorescence. The imaging periods were restricted to a constant period of time for each of the three NTHI strains to allow us to visually detect fluorescence differences amongst the three bacterial populations for each strain before we lost the ability to discriminate relative fluorescence by eye. Due to the inherent heterogeneity of NTHI (Duell et al., 2016), this translated to a total of 10 m for NTHI 86-028NP, 20 m for NTHI 1728, and 35 m for NTHI 1128. All assays were conducted at four separate times and on four separate days for each NTHI strain. Images and videos shown are representative of the independent assays. Microscopy data were processed and analyzed via NIS Elements software and arbitrary red fluorescence intensity in units (AFU) was analyzed with an NIS Elements General Analysis program (v. 5.42.03) (Nikon Instruments Inc.) and reported as arbitrary fluorescence units (AFU). To determine and normalize fluorescence, any background fluorescence at time 0 was removed for each individual bacterial population of each of the 3 NTHI isolates tested, and each strain was imaged separately for a set amount of time, e.g., 10, 20, or 35 min. Mean influx values in Table 1 were calculated from all raw data collected from replicate runs for each bacterial population and each strain.

**TABLE 1:** Migration of additional PMNs into the FOV was greatest in cultures of PMNs incubated with α-DNABII NRel of all three NTHI strains.

Bacterial population and NTHI strain	Number of PMNs in FOV at 0 min average ± SEM	Number of PMNs in FOV at 10 min average ± SEM	Influx of PMNs within 10 min average ± SEM
Planktonic 86-028NP	8 ± 2	22 ± 4	15 ± 4
NBfA 86-028NP	8 ± 4	42 ± 6	34 ± 3*
α-DNABII NRel 86-028NP	12 ± 2	63 ± 8	50 ± 9***,^†^
Planktonic 1128	8 ± 1	13 ± 2	6 ± 2
NBfA 1128	6 ± 2	26 ± 6	20 ± 5*
α-DNABII NRel 1128	8 ± 2	39 ± 3	31 ± 1**
Planktonic 1728	6 ± 1	16 ± 2	10 ± 2
NBfA 1728	9 ± 1	31 ± 3	22 ± 2**
α-DNABII NRel 1728	10 ± 2	41 ± 3	31 ± 1***,^†^

^a^“NBfA” refers to non-biofilm associated bacteria; “α-DNABII NRel” refers to bacteria newly released from biofilms via the action of a DNABII-directed monoclonal antibody. *^b^*Stars indicate significance between that population compared to planktonic bacteria within each individual NTHI strain. *^c^*Dagger indicates significance between that population compared to NBfA within each individual NTHI strain. ^d,^*,^†^*p* ≤ 0.05, **p ≤ 0.01, ***p ≤ 0.001.

### Flow cytometry

Bacterial solutions at 2e6 CFU/mL were sonicated for 2 m in a water bath sonicator to disrupt any aggregates, then 500 μL each bacterial solution was carefully added into wells of the 24-well plate such that 2e6 CFU/mL was added to wells that contained 500 μL of a 2e6 PMNs/mL solution. Samples were analyzed with a BD LSRFortessa flow cytometer (BD Biosciences, Franklin Lakes, NJ). Flow cytometry data were analyzed with FloJo software (v. 10.10.0) (FloJo, LLC, Ashland, OR). All flow cytometry was performed at least three separate times on at least three separate days for all populations of each NTHI strain.

### Endotoxin quantification

After NTHI populations were prepared and fluorescently labeled, the sodium bicarbonate buffer-bacterial suspensions were filter sterilized with a 0.22 μm syringe filter and the bacterial cell-free solutions were immediately subjected to endotoxin quantification via the Thermo Scientific™ Pierce™ Chromogenic Endotoxin Quant Kit (Thermo Fisher Scientific). Endotoxin quantification was performed per manufacturer’s instructions. Sterility was confirmed by spread plating on chocolate agar. Data represent the mean ± SEM of three biological replicates that were repeated on three separate days for each NTHI strain.

### DNA quantification

Quantification of relative extracellular DNA concentration within bacterial cell-free filter sterilized solutions in which each bacterial population had been suspended for incubation with human PMNs was performed via the Qubit dsDNA HS Assay Kit per manufacturer’s instructions (Thermo Fisher Scientific).

### Cytokine/chemokine analysis

Cytokine and chemokine assessment was performed via BD™ Cytometric Bead Array (CBA) Human Inflammatory Cytokine Cytometric Bead Array (CBA) – I Kit (BD Biosciences) or with BD™ Cytometric Bead Array (CBA) Human Flex Sets (BD Biosciences). To generate samples, the methodology we used to prepare NTHI populations for flow cytometry experiments was repeated. The 1 mL PMN-NTHI solution was collected after 10 m of co-incubation. Cytokine/chemokine analysis was performed immediately according to manufacturer’s instructions on an Accuri C6 flow cytometer (BD Biosciences) and data analyzed with FCAP Array™ Software v3.0.19.2091 (BD Biosciences). Data represent the mean ± SEM of three biological replicates that were repeated on three separate days for each NTHI strain.

### Statistics

Statistical tests were performed with GraphPad Prism 10 for all *in vitro* assays. Due to small sample sizes, a Shapiro-Wilk test for normality was performed on all data as well as a nonparametric Kruskal-Wallis test [with an uncorrected (i.e., non-parametric) Dunn’s test for multiple comparisons] to ensure that any stated statistical significance with parametric one-way ANOVAs (with Dunnett’s multiple comparison correction) (or lack thereof) were retained. Results from parametric testing are retained when data were normally distributed, and non-parametric test outcomes are reported for data that were not normally distributed. Data are presented as mean ± SEM and significance is shown as *p ≤ 0.05, **p ≤ 0.01, ***p ≤ 0.001, and ****p ≤ 0.0001. The number of biological replicates is defined within respective figure legends.

## Results

### Regardless of NTHI strain, PMNs incubated with pHrodo™-labeled NTHI populations demonstrated the greatest engulfment and NETosis activity with α-DNABII NTHI NRel

**FIGURE 1:**
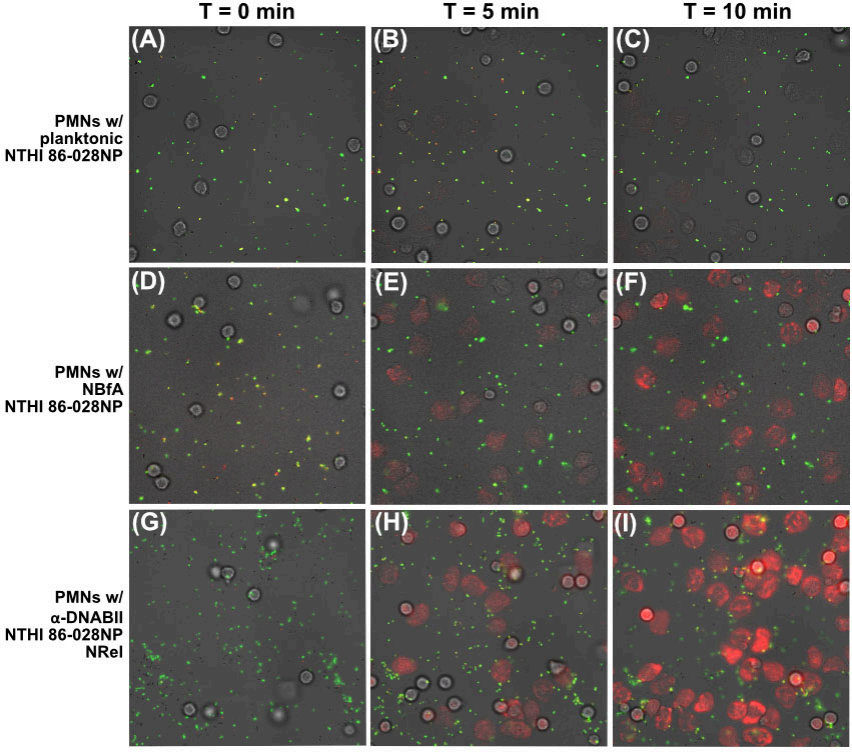
Relative PMN activity when incubated with three distinct populations of pHrodo™-labeled NTHI 86-028NP/pRSM2211. By timelapse microscopy, there was evidence of PMN-mediated uptake and NETosis when PMNs were incubated with planktonic **(A–C)**, NBfA **(D–F)**, or α-DNABII NRel **(G–I)** NTHI. Uptake, NETosis, and red fluorescence was visually greatest upon co-incubation of PMNs with α-DNABII NRel **(G–I)**. Results are representative of four separate assays. Similar results were also seen with two additional clinical isolates of NTHI (see Supplementary Figure 1). Collectively, these data indicated greatest PMN engulfment and NETosis activity was observed specifically with α-DNABII NRel NTHI.

When incubated with mid-log phase planktonically grown NTHI 86-028NP, all PMNs in the field of view (FOV) exhibited uptake activity throughout the 10 min imaging period as evidenced by PMNs that turned a light red color in addition to observing that a few PMNs began to exhibit NETosis activity, as assessed by visual loss of cellular membrane integrity (Figures 1A–C). When average arbitrary fluorescence intensity was quantified at the end of the imaging period, PMNs incubated with planktonic NTHI 86-028NP exhibited 98 arbitrary fluorescence units (AFU) (Table 2). When incubated with NBfA NTHI 86-028NP, all PMNs in the FOV engulfed NTHI and, in contrast to what we observed with planktonic NTHI 86-028NP, there was rapid (within 1–2 m) NETosis activity by the majority of PMNs that was evidenced by bright red fluorescence as demonstrated by approximately half of the PMNs in the FOV. This outcome yielded an average of 142 AFU (Figures 1D–F and Table 2). Intriguingly, PMNs incubated with α-DNABII NTHI 86-028NP NRel exhibited the most rapid uptake and NETosis activity, as well as the most striking red fluorescence with an average of 197 AFU which was significantly greater than that observed with either planktonic or NBfA populations (*p* ≤ 0.05–0.01) (Figures 1G–I and Table 2). In addition to images presented within Figure 1, relative PMN activity was documented by video microscopy (Supplementary Videos 1–3).

**TABLE 2:** Mean arbitrary red fluorescence units ± SEM emitted by PMNs when incubated with three NTHI strains and populations.

NTHI strain	Mean arbitrary fluorescence units (AFU) ± SEM		
	Planktonic	NBfA	α-DNABII NRel
86-028NP	98 ± 20	142 ± 23	197 ± 19**,^†^
1128	104 ± 12	163 ± 13	249 ± 17***,^†^
1728	104 ± 11	145 ± 7*	282 ± 8****,^†††^

^a^“NBfA” refers to non-biofilm associated bacteria; “α-DNABII NRel” refers to bacteria newly released from biofilms via the action of a DNABII-directed monoclonal antibody. *^b^*Stars indicate significance between that population compared to planktonic bacteria within each individual NTHI strain. *^c^*Dagger(s) indicate(s) significance between that population compared to NBfA within each individual NTHI strain. ^d,^*^†^*p* ≤ 0.05, **p ≤ 0.01, ***,^†⁣†⁣†^ *p* ≤ 0.001, ****p ≤ 0.0001.

When assayed with the second NTHI isolate, all PMNs in the FOV incubated with log phase planktonically grown NTHI 1128 exhibited rapid uptake activity throughout the 35 min imaging period wherein most PMNs exhibited a light red color. In addition, a few PMNs exhibited NETosis activity, which was similar to PMN activity with planktonic NTHI 86-028NP as reported above. PMNs incubated with NTHI 1128 that had been planktonically grown yielded an average of 104 AFU (Supplementary Figure 1A, panels a–c and Table 2). When incubated with NBfA NTHI 1128, all PMNs exhibited both uptake and rapid NETosis activity, with bright visual red fluorescence detected in most PMNs in the FOV for an average of 168 AFU (Supplementary Figure 1A, panels d–f and Table 2). When incubated with α-DNABII NTHI NRel of strain 1128, we again observed rapid uptake and NETosis activity within 2.5–3 min, as well as the most robust and striking visual red fluorescence with an average of 247 AFU which was significantly greater than that observed with either planktonic or NBfA populations (*p* ≤ 0.05–0.001) (Supplementary Figure 1A, panels g–i and Table 2). In addition to images presented within Supplementary Figure 1A, relative PMN activity was documented by video microscopy (Supplementary Videos 4–6).

When incubated with the third assessed NTHI strain, 1728, we observed engulfment and NETosis activity by PMNs incubated with mid-log phase planktonically grown NTHI 1728, and a few PMNs exhibited a light red color with an average of 109 AFU by the end of the imaging period (Supplementary Figure 1B, panels a–c and Table 2). When incubated with NBfA NTHI 1728, we observed both rapid PMN uptake and NETosis activity, and bright red fluorescence detected in approximately half of the PMNs in the FOV with an average of 143 AFU (Supplementary Figure 1B, panels d–f and Table 2). As seen with the other two NTHI isolates, PMNs incubated with α-DNABII NTHI 1728 NRel exhibited the most rapid uptake and NETosis activities as well as the greatest visual red fluorescence in approximately half the PMNs in the FOV, with an average of 287 AFU which was significantly greater than that observed with either planktonic or NBfA populations (*p* ≤ 0.05–0.0001) (Supplementary Figure 1B, panels g–i and Table 2). In addition to images presented within Supplementary Figure 1B, relative PMN activity was documented by video microscopy (Supplementary Videos 7–9).

### PMNs incubated with pHrodo™-labeled α-DNABII NTHI NRel exhibited the rightmost shift in red fluorescence by flow cytometry

**FIGURE 2:**
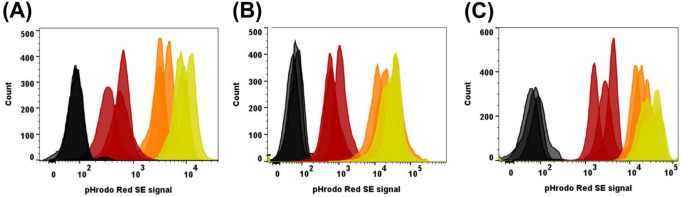
PMNs incubated with pHrodo™-labeled α-DNABII NTHI NRel exhibited the rightmost shift in red fluorescence by flow cytometry. Analysis of relative shifts in red fluorescence by PMNs incubated with pHrodo™-labeled planktonically grown, NBfA, or α-DNABII NTHI NRel via flow cytometry. PMNs without bacteria (black histograms) served as the negative control. Regardless of NTHI strain, PMNs incubated with planktonically grown (red histograms) NTHI 86-028NP **(A)**, NTHI 1128 **(B)**, or NTHI 1728 **(C)** exhibited a slight right shift in red fluorescence relative to the negative control. Relative red fluorescence was greater when incubated with NBfA of any NTHI isolate (orange histograms), however, overall greatest red fluorescence was seen in PMNs incubated with α-DNABII NTHI NRel (yellow histograms), thereby corroborating timelapse microscopy data. Results are representative of 3 independently run assays.

As our observations were primarily subjective to this point, to objectively detect and quantify relative red fluorescence by an additional methodology, we assessed the relative red fluorescence of PMNs collected after co-incubation with pHrodo™ Red SE-labeled planktonic, NBfA, or α-DNABII NRel NTHI populations via flow cytometry. In all cases, PMNs without bacteria served as the negative control and exhibited peak average red fluorescence of approximately 70 (Figure 2, black histograms). PMNs incubated with planktonically grown NTHI 86-028NP exhibited a slight right shift in peak red fluorescence, with a mean emission of approximately 5 × 10^2^ (Figure 2A, brown histograms), whereas those incubated with NBfA NTHI 86-028NP exhibited a greater right shift with a peak mean of approximately 2 × 10^3^ (Figure 2A, orange histograms). In keeping with data obtained by timelapse microscopy, the greatest and rightmost shift in red fluorescence was exhibited by PMNs incubated with α-DNABII NTHI NRel, wherein peak average red fluorescence was ∼1x10^4^ (Figure 2A, yellow histograms).

When incubated with planktonically grown NTHI 1128, PMNs exhibited a slight right shift in red fluorescence compared to PMNs without bacteria, with the mean peak at approximately 7 × 10^2^ (Figure 2B, brown histograms). When incubated with NBfA NTHI 1128, PMNs exhibited a mean red fluorescence of approximately 2 × 10^4^ (Figure 2B, orange histograms), and again the greatest right shift in peak average red fluorescence of approximately 3x10^4^ when incubated with α-DNABII NRel NTHI (Figure 2B, yellow histograms).

With the last of three clinical isolates assessed, PMNs incubated with planktonically grown NTHI 1728 exhibited a slight increase in red fluorescence relative to PMNs without bacteria, with an average peak of 2 × 10^3^ (Figure 2C, brown histograms). When incubated with NBfA NTHI 1728 the average red fluorescence occurred at ∼2 × 10^4^ (Figure 2C, orange histograms). The greatest right shift again occurred with PMNs incubated with α-DNABII NTHI 1728 NRel, wherein a peak average of approximately 4 × 10^4^ was obtained (Figure 2C, yellow histograms). While there was variability in relative placement of red fluorescence peaks, the rightmost shift in red fluorescence was consistently observed when PMNs were co-cultured with α-DNABII NTHI NRel of all three strains tested, which aligned well with what we observed by timelapse microscopy.

### Migration of additional PMNs into the FOV was greatest in cultures of PMNs incubated with α-DNABII NRel, regardless of NTHI strain

From our microscopy assays, in addition to PMN engulfment and NETosis activity, we observed that the relative movement of additional PMNs into the initial FOV, which were selected to ensure approximately the same number of PMNs at time 0 min for each NTHI strain and population tested, appeared distinct amongst the three bacterial populations. To investigate this further, we quantified the number of additional PMNs that migrated into a FOV when incubated with all three populations of the three NTHI strains.

When incubated with NTHI 86-028NP, the number of additional PMNs that entered the FOV within 10 min was: 15 ± 4 for planktonic NTHI, 34 ± 3 with NBfA which were statistically significant compared to planktonic (*p* = 0.02), and 50 ± 9 with α-DNABII NRel which was also statistically significant (*p* = 0.0008) (Table 1). For NTHI strain 1128, we observed similar results with the following number of additional PMNs having migrated into the FOV within 10 min when incubated with planktonic, NBfA, or NRel: 6 ± 2, 20 ± 5, and 31 ± 1, respectively, with the latter two values being statistically significant (*p* = 0.09, *p* = 0.004, respectively) compared to PMNs incubated with planktonic NTHI 1128 (Table 1). For NTHI 1728, 10 ± 2 PMNs entered the FOV within 10 min with planktonically grown, 22 ± 2 with NBfA, and 31 ± 1 with α-DNABII NRel. Again, these latter two values were statistically significant compared to planktonic (*p* = 0.003, *p* = 0.0002, respectively) (Table 1).

### Lag phase of α-DNABII NTHI NRel was not responsible for the observed differences in PMN activity amongst the three bacterial populations

**FIGURE 3:**
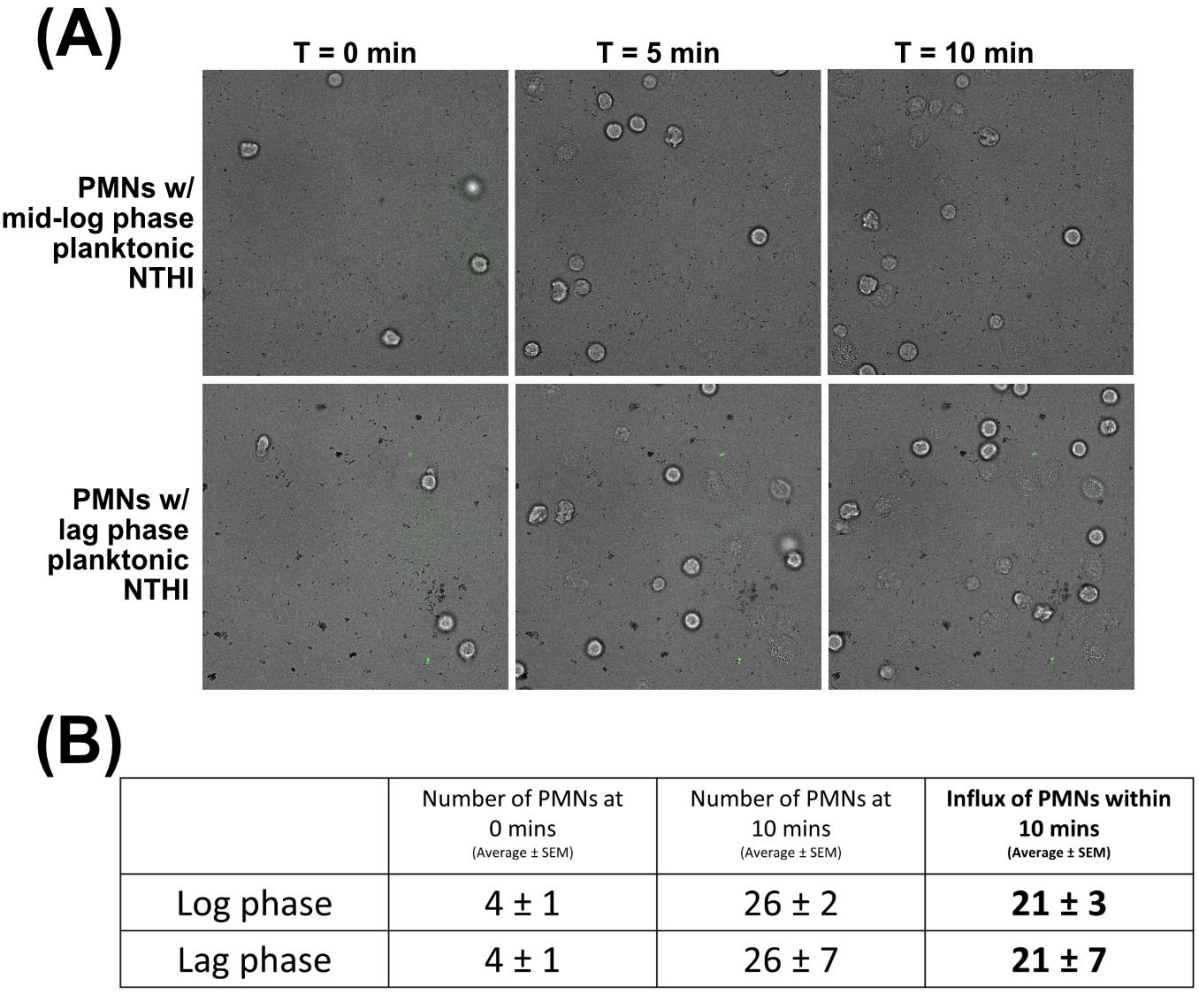
Lag phase of α-DNABII NTHI NRel was not responsible for the observed relative differences in PMN activity amongst the three populations of NTHI tested. As α-DNABII NTHI NRel exhibit significant upregulation of genes canonically indicative of lag phase bacterial growth, we wondered if PMN detection of lag phase alone was an inducer of greater PMN influx when incubated with α-DNABII NTHI NRel. These data showed no difference in relative PMN activity **(A)** or influx **(B)** when PMNs were incubated with NTHI 86-028NP grown planktonically to mid-log phase versus to lag phase, which indicated that lag phase of growth alone was not responsible for the significantly greater influx of PMNs seen with α-DNABII NTHI NRel. Results represent the average ± SEM of four independently run assays.

We recently reported that α-DNABII NTHI NRel are in lag phase as evidenced by their significantly upregulated expression of three genes canonically associated with this phase of bacterial growth (Wilbanks et al., 2023). As such, we wondered if perhaps characteristics of NTHI in this particular stage of growth might have played a role in the observed differences in relative PMN activity with this population of NTHI. To test this hypothesis, we recapitulated our timelapse microscopy assay and assessed relative PMN activity when incubated with mid-log phase planktonically grown NTHI 86-028NP or when incubated with lag phase planktonically grown NTHI 86-028NP. We observed no differences in PMN uptake or NETosis activity (Figure 3A), nor was there a difference in the average number of additional PMNs that migrated into the FOV (Figure 3B). These findings suggested that it was not only that α-DNABII NTHI NRel were specifically in lag phase which stimulated the greatest observed PMN activity.

### Relative differences in concentration of either NTHI endotoxin or DNA within supernatants in which NTHI populations were suspended when incubated with PMNs was also not responsible for the observed differences in relative PMN activity

**FIGURE 4:**
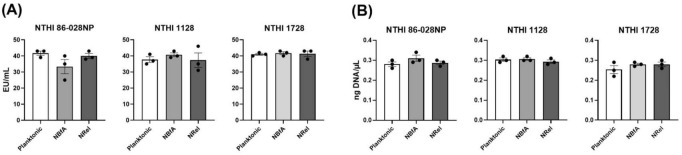
Differences in relative endotoxin or DNA levels were not responsible for the observed differences in relative PMN activity amongst the three tested NTHI populations. Regardless of NTHI strain or population assessed, there was no difference in quantified concentration of either bacterial endotoxin **(A)** or extracellular DNA (eDNA) **(B)** within bacterial cell-free fluids that contained washed and dual-fluorescently labeled planktonic, NBfA, or α-DNABII NTHI NRel, which suggested that neither endotoxin nor eDNA present within the fluids that had contained the bacterial populations when incubated with human PMNs were responsible for the observed significantly increased PMN influx when co-cultured with α-DNABII NTHI NRel. Endotoxin and eDNA results represent the average ± SEM of three independently run assays.

We next wondered whether either PMN uptake and NETosis activities, or the differences observed in terms of movement of additional PMNs into the FOV when PMNs were specifically incubated with α-DNABII NTHI NRel, might be due to differences in relative amounts of either bacterial endotoxin or DNA within preparations of the three NTHI populations. Therefore, we assayed for both of these NTHI-associated pathogen-associated molecular patterns (PAMPs) within the fluids in which the PMNs had been exposed, as each has been shown to induce PMN NETosis, as reported by Juneau et al. (2011). When we assessed relative levels of endotoxin, we found an average concentration of approximately 30–40 endotoxin units/mL within each of the planktonic, NBfA, and α-DNABII NTHI NRel populations for all three tested NTHI isolates which suggested that differences in relative endotoxin concentration were not responsible for notable differences observed in PMN activity (Figure 4A). We further determined the relative eDNA levels within the three population preparations for the three tested NTHI strains and found an average concentration of 0.3 ng DNA/μL in each (Figure 4B), which suggested that relative eDNA concentration was also not responsible for differences in observed PMN activity.

### The chemokine IL-8 was likely responsible for notable relative differences in migration of additional PMNs into the FOV when PMNs were incubated with α-DNABII NTHI NRel

**FIGURE 5:**
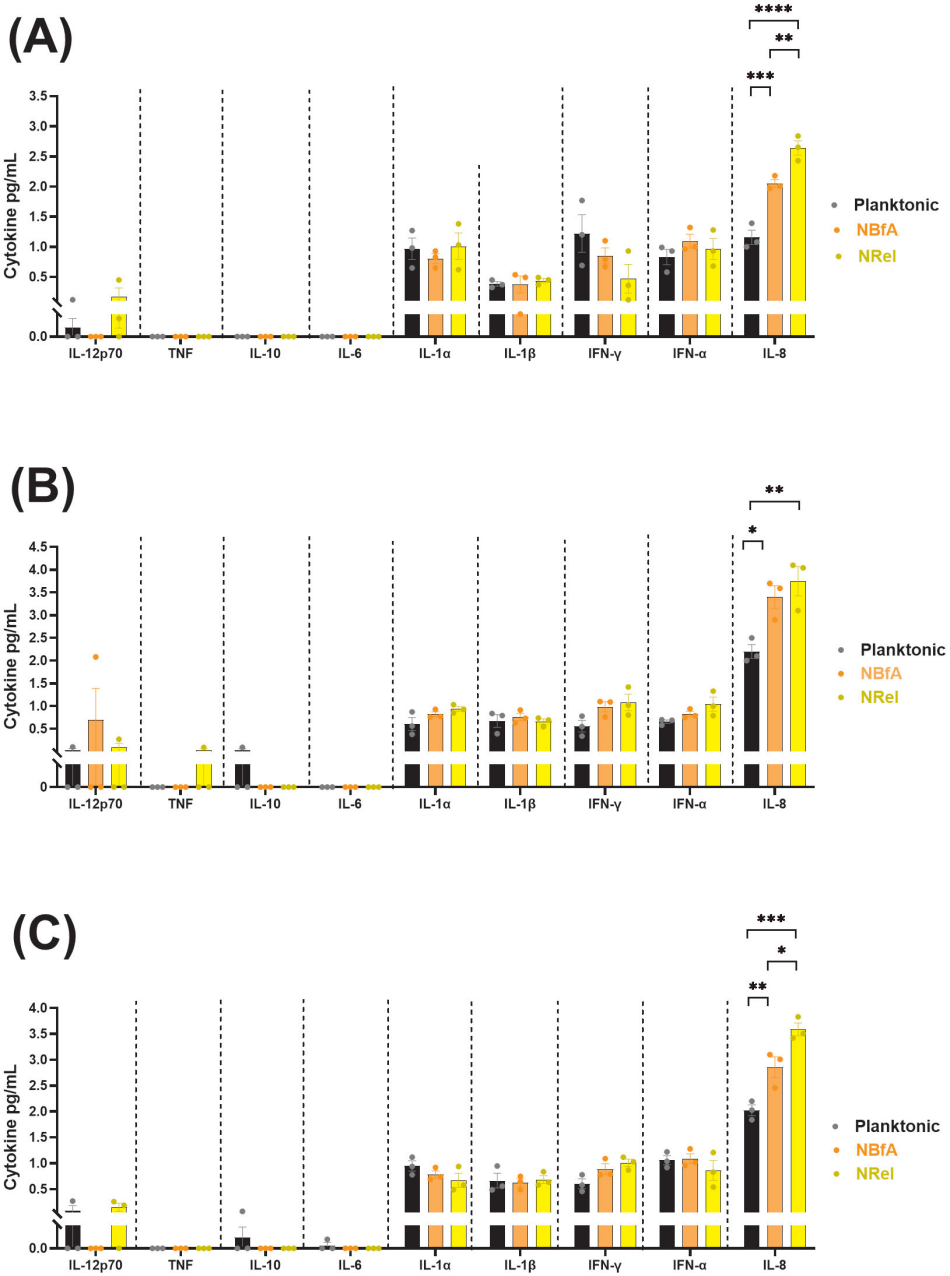
IL-8 was likely responsible for notable relative differences in migration of additional PMNs into the FOV when PMNs were incubated with α-DNABII NTHI NRel. Relative levels of human cytokines/chemokines produced by PMNs when incubated with planktonic, NBfA, or α-DNABII NTHI NRel were determined. Within 10 min of co-culture, levels of PMN-produced IL-8 were significantly increased when incubated with NBfA relative to planktonically grown NTHI for each of the 3 clinical NTHI isolates, 86-028NP **(A)**, 1128 **(B)**, or 1728 **(C)**. For NTHI strains 86-028NP **(A)** and 1728 **(C)**, the amount of IL-8 produced by PMNs incubated with α-DNABII NTHI NRel was also significantly greater compared to that when incubated with NBfA, results that were corroborated by microscopy and PMN migration datasets. However, regardless of NTHI strain tested, we consistently observed the greatest and most significantly increased levels of IL-8 produced by PMNs incubated with α-DNABII NTHI NRel relative to those incubated with planktonic NTHI. We observed no significant differences or trends for other assessed cytokines/chemokines. Increased levels of IL-8, a potent PMN-attracting chemokine, by PMNs exposed to α-DNABII NTHI NRel likely explains the observed and significant increase in average number of PMNs that entered the field of view in our microscopy assays. All results represent the average ± SEM of three independently run assays and statistical significance was determined via independent ordinary one-way ANOVAs. *p ≤ 0.05, **p ≤ 0.01, ***p ≤ 0.001, and ****p ≤ 0.0001.

We next considered whether the PMNs themselves might be signaling for the observed migration of additional PMNs into the FOV when they were incubated with α-DNABII NTHI NRel. To test this hypothesis, we assessed the media in which PMNs and NTHI populations were incubated for the presence of an array of human cytokines or chemokines after 10 min of co-incubation. We found no notable differences or trends in levels of IL-12p70, TNF, IL-10, or IL-6, regardless of the NTHI strain or population (Figures 5A–C). We also assessed levels of additional cytokines known to be both pro-inflammatory and neutrophil recruiters or activators: IL-1α, IL-1β, IFN-γ, and IFN-α (Chan et al., 2021). We observed increased levels of these latter cytokines, however, there were no observed trends in relative concentrations of these additional cytokines amongst the three NTHI populations of any of the three NTHI strains (Figures 5A–C). In contrast, levels of IL-8 were significantly increased in cultures of PMNs incubated with NBfA (average of 2.0–3.5 pg IL-8/mL) relative to those incubated with planktonic NTHI (average of 1.0–2.0 pg IL-8/mL), regardless of the NTHI strain assessed (*p* < 0.05–0.001) (Figures 5A–C). These results helped to validate our earlier timelapse microscopy-based observations in which we reported that significantly more PMNs entered the FOV when incubated with NBfA relative to those incubated with planktonically grown NTHI of all three strains assessed (see Table 1). Further, levels of IL-8 were also significantly increased and, in fact the greatest overall, when PMNs were incubated with α-DNABII NTHI NRel (average of 2.5–4.0 pg IL-8/mL) relative to those incubated with planktonic NTHI (*p* < 0.01–0.0001), regardless of the NTHI strain, which also aligned well with our microscopy and quantification of additional PMNs that migrated into the FOV datasets. Further, significantly more PMNs entered the FOV when incubated with α-DNABII NTHI 86-028NP or 1728 NRel relative to when incubated with NBfA of either strain (*p* < 0.01 and 0.05, respectively) (Figure 5A–C). We did not observe similar significant differences in IL-8 levels between α-DNABII NTHI 1128 NRel and NBfA (*p* = 0.36) (Figure 5B), which was similarly corroborated by our video microscopy data and quantification of additional migrated PMNs (Table 1).

## Discussion

There is an urgent need for highly effective novel therapeutics, and ideally preventative strategies, for better management of chronic and recurrent biofilm-related diseases, as they are notoriously recalcitrant to clearance by antibiotics and effectors of the host immune system (Roilides et al., 2015; Grooters et al., 2024). One strategy to address these issues is to actively release biofilm-resident bacteria from their protective matrix such that they are now more readily accessible to both conventional antibiotics and immune effectors. To that end, we have focused on structurally essential proteins within the biofilm matrix, the bacterial DNABII proteins (Novotny et al., 2016, 2020; Rogers et al., 2022), which bind at the vertices of crossed-strands of extracellular DNA to stabilize the biofilm matrix (Goodman et al., 2011). When a monoclonal antibody directed against the immunoprotective DNA-binding “tips” of a DNABII protein is incubated with a bacterial biofilm formed individually by any of 24 distinct genera, each member of the ESKAPEE pathogens, or with polymicrobial biofilms, the biofilm collapses within minutes (Novotny et al., 2016, 2019; Jurcisek et al., 2022; Kurbatfinski et al., 2022, Kurbatfinski et al., 2023a,b; Wilbanks et al., 2023; Rhodes et al., 2024). These rapidly released formerly biofilm-resident pathogens are now significantly more susceptible to killing by antibiotics and antimicrobial peptides *in vitro* (Novotny et al., 2016; Jurcisek et al., 2022; Kurbatfinski et al., 2022, Kurbatfinski et al., 2023a,b; Wilbanks et al., 2023; Rhodes et al., 2024).

Further, we’ve now demonstrated pre-clinical efficacy in four models of biofilm infections after treatment with this DNABII-directed monoclonal (Novotny et al., 2016, 2021; Freire et al., 2017; Jurcisek et al., 2025). Importantly, these outcomes were achieved without use of any co-delivered antibiotics, which highlighted the primary role of innate immune effectors. We found that in either the rat oral cavity, chinchilla middle ear or mouse lungs the mucosa appeared to rapidly return to homeostatic states in α-DNABII-treated animals (Novotny et al., 2016, 2021; Freire et al., 2017; Jurcisek et al., 2025). When we tested the role of innate immune effectors *in vitro*, we indeed found support for this hypothesis, as NTHI newly released from biofilms via α-DNABII were significantly more susceptible to killing by antimicrobial peptides and human PMNs (Wilbanks et al., 2023; Kurbatfinski et al., 2025). However, we did not yet know the full complement of differences in overall PMN activity when PMNs encountered newly released NTHI, as they would have in the pre-clinical efficacy models described above.

Data presented here revealed that PMN activity was indeed notably different when exposed to newly released NTHI. Whereas PMNs demonstrated uptake and NETosis with all tested populations of NTHI, they were nonetheless able to discriminate between NTHI grown planktonically *versus* those present in fluid above a biofilm *versus* those rapidly released from biofilm residence via the DNABII-targeted monoclonal. Further, while the overall trends of activity amongst the tested bacterial populations were highly similar for all three clinical isolates of NTHI assessed herein, there was nonetheless some slight variability in PMN activity which is likely due to vast heterogeneity of NTHI, as has been previously documented for these isolates both *in vitro* as well as when characterized in pre-clinical models (Bakaletz et al., 1988; Sirakova et al., 1994; DeMaria et al., 1996; Miyamoto and Bakaletz, 1996; Bakaletz et al., 1997; Novotny and Bakaletz, 2003). Regardless of any slight differences observed amongst NTHI strains within data obtained via timelapse microscopy, flow cytometry, and chemokine analysis as presented here, we nonetheless observed highly similar overall patterns when PMNs were incubated with planktonically grown NTHI versus those recovered from the fluids above a biofilm or those newly released from biofilm residence by the action of α-DNABII, regardless of the strain assessed. It should be noted that whereas we did test three clinical isolates of NTHI, each with their own known inherent heterogeneity in the work presented here, these are nonetheless all isolates recovered from children with chronic otitis media which is therein an acknowledged potential limitation.

Interestingly, it is important to note that PMN activity with NBfA NTHI tended to be more similar to that with α-DNABII NTHI NRel than it was to PMN activity with NTHI grown planktonically in all ways assessed here. The NBfA population is likely an isogenic but heterogeneous mix of phenotypically variable cells (Spratt and Lane, 2022) that includes both NTHI that are simply growing in the fluids above the biofilm as well as those that have come off the biofilm as a result of natural biofilm remodeling (Kostakioti et al., 2013). Given that bacteria growing planktonically in nutrient-rich media do not exist in nature, we used the NBfA population to attempt to more closely represent those bacteria found in a disease site that are within fluids in close proximity to the pathogenic biofilm but not resident within it (e.g., synovial joint fluids, middle ear effusions, lung aspirates, etc.). The notable PMN activity we report here with the NBfA population was intriguing as this is likely a very heterogeneous mix of NTHI that accumulated in the medium that overlayed the biofilm in the 2 h incubation period described here. This population is believed to be a mix of bacteria which have perhaps come off the biofilm due to natural remodeling, include non-adherent bacteria that were not washed off prior to the 2 h incubation period and even those that may have divided during planktonic growth in this period of time. That PMNs were quite active with the NBfA population may help explain why fluids recovered from clinical sites of bacterial infection, wherein biofilms are implicated, are often culture negative despite the continued presence of a pathogenic biofilm in the same site. Whereas these clinical fluids may contain viable but not culturable bacteria, this sterile status may also be reflective of enhanced PMN activity against these NBfA-like bacteria, as well as that of other immune effectors, that are otherwise ineffective when these disease-associated pathogens are resident within the biofilm (Fux et al., 2006; Schulz et al., 2021).

That healthy human PMNs were able to distinguish amongst the three tested populations of NTHI was not explained by relative differences in levels of endotoxin (i.e., lipooligosaccharide), eDNA, or the characteristic lag phase of newly released NTHI (Wilbanks et al., 2023). There was, however, a significant difference in levels of PMN-produced interleukin (IL)-8 detected when human PMNs were incubated with either NBfA or NRel compared to planktonic bacteria. IL-8 is a potent PMN-recruiting chemokine with additional important roles in activation of PMNs to release granule contents, generate superoxide, and increase expression of adhesion molecules (Bickel, 1993; Matsushima et al., 2022). Intra-PMN signaling via IL-8 when incubated with newly released NTHI is another likely mechanism by which this important innate immune effector contributed to the outcomes we reported to-date in four pre-clinical models of biofilm disease resolution wherein we targeted the DNABII proteins (Novotny et al., 2016; Freire et al., 2017; Novotny et al., 2021; Jurcisek et al., 2025). It is important to note that whereas both planktonic and biofilm-resident NTHI have mechanisms to evade PMN killing (Hong et al., 2009; Juneau et al., 2011), these two populations of NTHI have notably different phenotypes from those that have been freshly dispersed from bacterial biofilms via a number of mechanisms as has been reported for multiple pathogens (Rollet et al., 2009; Chua et al., 2014; Barraud et al., 2015; Guilhen et al., 2016; Uppuluri et al., 2018; Kalia and Sauer, 2024; Sahu et al., 2025). Further, both we and others have shown that this distinct phenotype and transcriptome is evident within minutes of dispersal from a biofilm (Mokrzan et al., 2020; Wilbanks et al., 2023; Kalia and Sauer, 2024).

Thereby, we posit that the enhanced activity we report here when human PMNs encountered NRel populations of NTHI, as well as earlier (Wilbanks et al., 2023), and in pre-clinical models (Novotny et al., 2016, 2021; Freire et al., 2017; Jurcisek et al., 2025), is likely due to the specific manner of active release mediated by the anti-DNABII monoclonal antibody combined with the distinct phenotype of this newly released population. Intriguingly, *Pseudomonas aeruginosa* freshly dispersed from a biofilm as induced via the action of nitric oxide and glutamate was shown capable of significantly evading engulfment by macrophages (Kalia and Sauer, 2024), and the potential for increased virulence has been reported for other bacteria actively dispersed from a biofilm via a programmed pathway (Fleming and Rumbaugh, 2018; Murray et al., 2025). Taken together, it appears likely that there is yet another aspect of phenotypic variability between freshly dispersed bacteria (e.g., as induced by either native or active induction to mediate dispersal via a programmed pathway) *versus* that observed in newly released bacteria (e.g., as mediated by rapid collapse of the biofilm as described here), the latter of which does not allow for bacterial adaptation prior to exit from the protective biofilm. Nonetheless, whereas fresh actively dispersed bacterial cells and those newly but passively released (due to an external disturbance that physically or enzymatically destroys the biofilm matrix or detaches it) do share some phenotypic similarities (Murray et al., 2025), importantly no evidence of enhanced virulence has been observed for newly released bacteria (Novotny et al., 2016, 2021; Freire et al., 2017; Redman et al., 2021; Jurcisek et al., 2025). What we do not yet know is whether any of the described phenotypes are specific to rapid release by anti-DNABII or if other forms of rapid release will induce the same outcome, however, this is an active area of investigation for our laboratories.

Collectively, new data described here provide additional mechanistic insights as to how NTHI newly released from biofilms via the DNABII-targeted monoclonal are likely rapidly and effectively cleared *in vivo*. It is worth noting that treatment with this targeted monoclonal may also benefit from either carefully titrated delivery and/or supplemental antibiotic treatment, depending on the host’s immune status, nature of the disease, and location of the infection, in order to ensure that release of biofilm-resident bacteria does not produce an overwhelming inflammatory response or wherein released bacteria could travel to other parts of the body and/or potentially cause septicemia (Koo et al., 2017; Fleming and Rumbaugh, 2018). However, we nonetheless believe this monoclonal antibody to be a potentially widely applicable therapeutic option across a range of diverse diseases given that it targets universal and structurally essential proteins of the biofilm matrix as evidenced by data which shows that it can rapidly and significantly disrupt biofilms formed by 24 diverse genera of pathogens to date (Mokrzan et al., 2018, 2020; Kurbatfinski et al., 2022, Kurbatfinski et al., 2023a,b; Wilbanks et al., 2023). This specifically targeted monoclonal is currently in clinical trials [NCT05629741; NCT06159725] wherein we aspire for a positive outcome as a result of similar rapid eradication of pathogenic biofilms with clearance of bacteria once released from their protective biofilm matrix. In a world where antibiotic resistance and immune evasion lend numerous advantages to bacteria resident within pathogenic biofilms, it is our hope that this monoclonal antibody-based approach, designed to be pathogen-agnostic, may provide a widely effective and powerful therapeutic option.

## Data Availability

The original contributions presented in this study are included in this article/Supplementary material, further inquiries can be directed to the corresponding author.

## References

[B1] Bakaletz L. O. Leake E. R. Billy J. M. Kaumaya P. T. (1997). Relative immunogenicity and efficacy of two synthetic chimeric peptides of fimbrin as vaccinogens against nasopharyngeal colonization by nontypeable *Haemophilus influenzae* in the chinchilla. *Vaccine* 15 955–961. 10.1016/s0264-410x(96)00298-8 9261941

[B2] Bakaletz L. O. Tallan B. M. Hoepf T. DeMaria T. F. Birck H. G. Lim D. J. (1988). Frequency of fimbriation of nontypable *Haemophilus influenzae* and its ability to adhere to chinchilla and human respiratory epithelium. *Infect. Immun.* 56 331–335. 10.1128/iai.56.2.331-335.1988 2892792 PMC259284

[B3] Barraud N. Kjelleberg S. Rice S. A. (2015). Dispersal from microbial biofilms. *Microbiol. Spectr.* 3:MB-0015-2014. 10.1128/microbiolspec.MB-0015-2014. 27337281

[B4] Bickel M. (1993). The role of interleukin-8 in inflammation and mechanisms of regulation. *J. Periodontol.* 64(5 Suppl.), 456–460. 8315568

[B5] Boisvert A. A. Cheng M. P. Sheppard D. C. Nguyen D. (2016). Microbial biofilms in pulmonary and critical care diseases. *Ann. Am. Thorac Soc.* 13 1615–1623. 10.1513/AnnalsATS.201603-194FR 27348071 PMC5059503

[B6] Buzzo J. R. Devaraj A. Gloag E. S. Jurcisek J. A. Robledo-Avila F. Kesler T. (2021). Z-form extracellular DNA is a structural component of the bacterial biofilm matrix. *Cell* 184 5740–5758 e17. 10.1016/j.cell.2021.10.010 34735796 PMC8595767

[B7] Chambers J. R. Cherny K. E. Sauer K. (2017). Susceptibility of *Pseudomonas aeruginosa* dispersed cells to antimicrobial agents is dependent on the dispersion cue and class of the antimicrobial agent used. *Antimicrob. Agents Chemother.* 61:e00846-17. 10.1128/aac.00846-17 28971863 PMC5700346

[B8] Chan L. Karimi N. Morovati S. Alizadeh K. Kakish J. E. Vanderkamp S. (2021). The roles of neutrophils in cytokine storms. *Viruses* 13:2318. 10.3390/v13112318 34835125 PMC8624379

[B9] Chua S. L. Liu Y. Yam J. K. Chen Y. Vejborg R. M. Tan B. G. (2014). Dispersed cells represent a distinct stage in the transition from bacterial biofilm to planktonic lifestyles. *Nat. Commun.* 5:4462. 10.1038/ncomms5462 25042103

[B10] DeMaria T. F. Murwin D. M. Leake E. R. (1996). Immunization with outer membrane protein P6 from nontypeable *Haemophilus influenzae* induces bactericidal antibody and affords protection in the chinchilla model of otitis media. *Infect. Immun.* 64 5187–5192. 10.1128/iai.64.12.5187-5192.1996 8945564 PMC174506

[B11] Devaraj A. Buzzo J. Rocco C. J. Bakaletz L. O. Goodman S. D. (2018). The DNABII family of proteins is comprised of the only nucleoid associated proteins required for nontypeable *Haemophilus influenzae* biofilm structure. *Microbiologyopen* 7:e00563. 10.1002/mbo3.563 29230970 PMC6011942

[B12] Dey D. Nagaraja V. Ramakumar S. (2017). Structural and evolutionary analyses reveal determinants of DNA binding specificities of nucleoid-associated proteins HU and IHF. *Mol. Phylogenet. Evol.* 107 356–366. 10.1016/j.ympev.2016.11.014 27894997

[B13] Di Domenico E. G. Oliva A. Guembe M. (2022). The current knowledge on the pathogenesis of tissue and medical device-related biofilm infections. *Microorganisms* 10:1259. 10.3390/microorganisms10071259 35888978 PMC9322301

[B14] Duell B. L. Su Y. C. Riesbeck K. (2016). Host-pathogen interactions of nontypeable *Haemophilus influenzae*: From commensal to pathogen. *FEBS Lett.* 590 3840–3853. 10.1002/1873-3468.12351 27508518

[B15] Fleming D. Rumbaugh K. (2018). The consequences of biofilm dispersal on the host. *Sci. Rep.* 8:10738. 10.1038/s41598-018-29121-2 30013112 PMC6048044

[B16] Freire M. O. Devaraj A. Young A. Navarro J. B. Downey J. S. Chen C. (2017). A bacterial-biofilm-induced oral osteolytic infection can be successfully treated by immuno-targeting an extracellular nucleoid-associated protein. *Mol. Oral Microbiol.* 32 74–88. 10.1111/omi.12155 26931773 PMC5010536

[B17] Fux C. A. Quigley M. Worel A. M. Post C. Zimmerli S. Ehrlich G. (2006). Biofilm-related infections of cerebrospinal fluid shunts. *Clin. Microbiol. Infect.* 12 331–337. 10.1111/j.1469-0691.2006.01361.x 16524409

[B18] Goodman S. D. Obergfell K. P. Jurcisek J. A. Novotny L. A. Downey J. S. Ayala E. A. (2011). Biofilms can be dispersed by focusing the immune system on a common family of bacterial nucleoid-associated proteins. *Mucosal Immunol.* 4 625–637. 10.1038/mi.2011.27 21716265

[B19] Goodwine J. Gil J. Doiron A. Valdes J. Solis M. Higa A. (2019). Pyruvate-depleting conditions induce biofilm dispersion and enhance the efficacy of antibiotics in killing biofilms in vitro and in vivo. *Sci. Rep.* 9:3763. 10.1038/s41598-019-40378-z 30842579 PMC6403282

[B20] Grooters K. E. Ku J. C. Richter D. M. Krinock M. J. Minor A. Li P. (2024). Strategies for combating antibiotic resistance in bacterial biofilms. *Front. Cell Infect. Microbiol.* 14:1352273. 10.3389/fcimb.2024.1352273 38322672 PMC10846525

[B21] Guilhen C. Charbonnel N. Parisot N. Gueguen N. Iltis A. Forestier C. (2016). Transcriptional profiling of *Klebsiella pneumoniae* defines signatures for planktonic, sessile and biofilm-dispersed cells. *BMC Genom.* 17:237. 10.1186/s12864-016-2557-x 26979871 PMC4791964

[B22] Hong W. Juneau R. A. Pang B. Swords W. E. (2009). Survival of bacterial biofilms within neutrophil extracellular traps promotes nontypeable *Haemophilus influenzae* persistence in the chinchilla model for otitis media. *J. Innate Immun.* 1 215–224. 10.1159/000205937 20375579 PMC6951045

[B23] Juneau R. A. Pang B. Weimer K. E. Armbruster C. E. Swords W. E. (2011). Nontypeable *Haemophilus influenzae* initiates formation of neutrophil extracellular traps. *Infect Immun.* 79 431–438. 10.1128/IAI.00660-10 20956567 PMC3019868

[B24] Jurcisek J. A. Hofer L. K. Goodman S. D. Bakaletz L. O. (2022). Monoclonal antibodies that target extracellular DNABII proteins or the type IV pilus of nontypeable *Haemophilus influenzae* (NTHI) worked additively to disrupt 2-genera biofilms. *Biofilm* 4:100096. 10.1016/j.bioflm.2022.100096 36532267 PMC9747592

[B25] Jurcisek J. A. Kurbatfinski N. Wilbanks K. Q. Rhodes J. D. Goodman S. D. Bakaletz L. O. (2025). *Mycobacterium abscessus* biofilm cleared from murine lung by monoclonal antibody against bacterial DNABII proteins. *J. Cyst. Fibros* 24 374–381. 10.1016/j.jcf.2025.01.013 39919951

[B26] Kalia M. Sauer K. (2024). Distinct transcriptome and traits of freshly dispersed *Pseudomonas aeruginosa* cells. *mSphere* 9:e0088424. 10.1128/msphere.00884-24 39601567 PMC11656770

[B27] Koo H. Allan R. N. Howlin R. P. Stoodley P. Hall-Stoodley L. (2017). Targeting microbial biofilms: Current and prospective therapeutic strategies. *Nat. Rev. Microbiol.* 15 740–755. 10.1038/nrmicro.2017.99 28944770 PMC5685531

[B28] Kostakioti M. Hadjifrangiskou M. Hultgren S. J. (2013). Bacterial biofilms: Development, dispersal, and therapeutic strategies in the dawn of the postantibiotic era. *Cold Spring Harb. Perspect. Med.* 3:a010306. 10.1101/cshperspect.a010306 23545571 PMC3683961

[B29] Kragh K. N. Tolker-Nielsen T. Lichtenberg M. (2023). The non-attached biofilm aggregate. *Commun. Biol.* 6:898. 10.1038/s42003-023-05281-4 37658117 PMC10474055

[B30] Kurbatfinski N. Goodman S. D. Bakaletz L. O. (2022). A humanized monoclonal antibody potentiates killing of diverse biofilm-forming respiratory tract pathogens by antibiotics. *Antimicrob. Agents Chemother.* 66 e0187721. 10.1128/aac.01877-21 35007137 PMC8923185

[B31] Kurbatfinski N. Hill P. J. Tobin N. Kramer C. N. Wickham J. Goodman S. D. (2023a). Disruption of nontuberculous mycobacteria biofilms induces a highly vulnerable to antibiotic killing phenotype. *Biofilm* 6:100166. 10.1016/j.bioflm.2023.100166 38078059 PMC10698573

[B32] Kurbatfinski N. Kramer C. N. Goodman S. D. Bakaletz L. O. (2023b). ESKAPEE pathogens newly released from biofilm residence by a targeted monoclonal are sensitized to killing by traditional antibiotics. *Front. Microbiol.* 14:1202215. 10.3389/fmicb.2023.1202215 37564292 PMC10410267

[B33] Kurbatfinski N. Jurscisek J. A. Wilbanks K. Q. Goodman S. D. Bakaletz L. O. (2025). Respiratory tract antimicrobial peptides more effectively killed multiple methicillin-resistant *Staphylococcus aureus* and nontypeable *Haemophilus influenzae* isolates after disruption from biofilm residence. *Microbiol. Spectr.* 13:e0306624. 10.1128/spectrum.03066-24 40530663 PMC12323582

[B34] Lee W. L. Harrison R. E. Grinstein S. (2003). Phagocytosis by neutrophils. *Microbes Infect.* 5 1299–1306. 10.1016/j.micinf.2003.09.014 14613773

[B35] Li Y. Petrova O. E. Su S. Lau G. W. Panmanee W. Na R. (2014). BdlA, DipA and induced dispersion contribute to acute virulence and chronic persistence of *Pseudomonas aeruginosa*. *PLoS Pathog* 10:e1004168. 10.1371/journal.ppat.1004168 24901523 PMC4047105

[B36] Liew P. X. Kubes P. (2019). The neutrophil’s role during health and disease. *Physiol. Rev.* 99 1223–1248. 10.1152/physrev.00012.2018 30758246

[B37] Marks L. R. Davidson B. A. Knight P. R. Hakansson A. P. (2013). Interkingdom signaling induces *Streptococcus pneumoniae* biofilm dispersion and transition from asymptomatic colonization to disease. *mBio* 4:e0043813. 10.1128/mBio.00438-13 23882016 PMC3735180

[B38] Mason K. M. Munson R. S. Jr. Bakaletz L. O. (2003). Nontypeable *Haemophilus influenzae* gene expression induced *in vivo* in a chinchilla model of otitis media. *Infect Immun.* 71 3454–3462. 10.1128/iai.71.6.3454-3462.2003 12761130 PMC155704

[B39] Matsushima K. Yang D. Oppenheim J. J. (2022). Interleukin-8: An evolving chemokine. *Cytokine* 153:155828. 10.1016/j.cyto.2022.155828 35247648

[B40] Mirghani R. Saba T. Khaliq H. Mitchell J. Do L. Chambi L. (2022). Biofilms: Formation, drug resistance and alternatives to conventional approaches. *AIMS Microbiol.* 8 239–277. 10.3934/microbiol.2022019 36317001 PMC9576500

[B41] Miyamoto N. Bakaletz L. O. (1996). Selective adherence of non-typeable *Haemophilus influenzae* (NTHi) to mucus or epithelial cells in the chinchilla eustachian tube and middle ear. *Microb Pathog* 21 343–356. 10.1006/mpat.1996.0067 8938642

[B42] Mokrzan E. M. Ahearn C. P. Buzzo J. R. Novotny L. A. Zhang Y. Goodman S. D. (2020). Nontypeable *Haemophilus influenzae* newly released (NRel) from biofilms by antibody-mediated dispersal versus antibody-mediated disruption are phenotypically distinct. *Biofilm* 2:100039. 10.1016/j.bioflm.2020.100039 33447823 PMC7798465

[B43] Mokrzan E. M. Novotny L. A. Brockman K. L. Bakaletz L. O. (2018). Antibodies against the majority subunit (PilA) of the type IV pilus of nontypeable *Haemophilus influenzae* disperse *Moraxella catarrhalis* from a dual-species biofilm. *mBio* 9:e02423-18. 10.1128/mBio.02423-18 30538189 PMC6299487

[B44] Murray R. K. Martin A. E. Zipkowitz S. Jahan N. Davis T. D. Redman W. K. (2025). alpha-amylase-mediated antibiotic degradation and sequestration in *Pseudomonas aeruginosa* biofilm therapy. *Antibiotics* 14:941. 10.3390/antibiotics14090941 41009919 PMC12466716

[B45] Novotny L. A. Bakaletz L. O. (2003). The fourth surface-exposed region of the outer membrane protein P5-homologous adhesin of nontypable *Haemophilus influenzae* is an immunodominant but nonprotective decoying epitope. *J. Immunol.* 171 1978–1983. 10.4049/jimmunol.171.4.1978 12902501

[B46] Novotny L. A. Chiang T. Goodman S. D. Elmaraghy C. A. Bakaletz L. O. (2021). Humanized anti-DNABII Fab fragments plus ofloxacin eradicated biofilms in experimental otitis media. *Laryngoscope* 131 E2698–E2704. 10.1002/lary.29497 33666254 PMC8609234

[B47] Novotny L. A. Goodman S. D. Bakaletz L. O. (2019). Redirecting the immune response towards immunoprotective domains of a DNABII protein resolves experimental otitis media. *NPJ Vaccines* 4:43. 10.1038/s41541-019-0137-1 31632744 PMC6791836

[B48] Novotny L. A. Goodman S. D. Bakaletz L. O. (2020). Targeting a bacterial DNABII protein with a chimeric peptide immunogen or humanised monoclonal antibody to prevent or treat recalcitrant biofilm-mediated infections. *EBioMedicine* 59:102867. 10.1016/j.ebiom.2020.102867 32651162 PMC7502671

[B49] Novotny L. A. Jurcisek J. A. Goodman S. D. Bakaletz L. O. (2016). Monoclonal antibodies against DNA-binding tips of DNABII proteins disrupt biofilms in vitro and induce bacterial clearance in vivo. *EBioMedicine* 10 33–44. 10.1016/j.ebiom.2016.06.022 27342872 PMC5006588

[B50] Petrova O. E. Sauer K. (2016). Escaping the biofilm in more than one way: Desorption, detachment or dispersion. *Curr. Opin. Microbiol.* 30 67–78. 10.1016/j.mib.2016.01.004 26826978 PMC4821722

[B51] Redman W. K. Welch G. S. Williams A. C. Damron A. J. Northcut W. O. Rumbaugh K. P. (2021). Efficacy and safety of biofilm dispersal by glycoside hydrolases in wounds. *Biofilm* 3:100061. 10.1016/j.bioflm.2021.100061 34825176 PMC8605310

[B52] Rhodes J. D. Devaraj A. Robledo-Avila F. Balu S. Mashburn-Warren L. Buzzo J. R. (2024). Noninflammatory 97-amino acid high mobility group box 1 derived polypeptide disrupts and prevents diverse biofilms. *EBioMedicine* 107:105304. 10.1016/j.ebiom.2024.105304 39182358 PMC11385066

[B53] Rogers J. V. Hall V. L. McOsker C. C. (2022). Crumbling the castle: Targeting DNABII proteins for collapsing bacterial biofilms as a therapeutic approach to treat disease and combat antimicrobial resistance. *Antibiotics* 11:104. 10.3390/antibiotics11010104 35052981 PMC8773079

[B54] Roilides E. Simitsopoulou M. Katragkou A. Walsh T. J. (2015). How biofilms evade host defenses. *Microbiol. Spectr.* 3 1–10. 10.1128/microbiolspec.MB-0012-2014 26185085

[B55] Rollet C. Gal L. Guzzo J. (2009). Biofilm-detached cells, a transition from a sessile to a planktonic phenotype: A comparative study of adhesion and physiological characteristics in *Pseudomonas aeruginosa*. *FEMS Microbiol. Lett.* 290 135–142. 10.1111/j.1574-6968.2008.01415.x 19054076

[B56] Rumbaugh K. P. Sauer K. (2020). Biofilm dispersion. *Nat. Rev. Microbiol.* 18 571–586. 10.1038/s41579-020-0385-0 32533131 PMC8564779

[B57] Sahu A. Jain S. Junghare M. Mishra A. Ruhal R. (2025). Biofilm-dispersal patterns in ESKAPE pathogens. *Arch. Microbiol.* 207:194. 10.1007/s00203-025-04394-0 40643714

[B58] Schulz P. Dlaska C. E. Perka C. Trampuz A. Renz N. (2021). Preoperative synovial fluid culture poorly predicts the pathogen causing periprosthetic joint infection. *Infection* 49 427–436. 10.1007/s15010-020-01540-2 33141393 PMC8159841

[B59] Shrestha L. Fan H. M. Tao H. R. Huang J. D. (2022). Recent strategies to combat biofilms using antimicrobial agents and therapeutic approaches. *Pathogens* 11:292. 10.3390/pathogens11030292 35335616 PMC8955104

[B60] Sirakova T. Kolattukudy P. E. Murwin D. Billy J. Leake E. Lim D. (1994). Role of fimbriae expressed by nontypeable *Haemophilus influenzae* in pathogenesis of and protection against otitis media and relatedness of the fimbrin subunit to outer membrane protein A. *Infect Immun.* 62 2002–2020. 10.1128/iai.62.5.2002-2020.1994 7909539 PMC186460

[B61] Spratt M. R. Lane K. (2022). Navigating environmental transitions: The role of phenotypic variation in bacterial responses. *mBio* 13:e0221222. 10.1128/mbio.02212-22 36259726 PMC9765552

[B62] Swinger K. K. Rice P. A. (2004). IHF and HU: Flexible architects of bent DNA. *Curr. Opin. Struct. Biol.* 14 28–35. 10.1016/j.sbi.2003.12.003 15102446

[B63] Uppuluri P. Acosta Zaldivar M. Anderson M. Z. Dunn M. J. Berman J. Lopez Ribot J. L. (2018). *Candida albicans* dispersed cells are developmentally distinct from biofilm and planktonic cells. *mBio* 9:e01338-18. 10.1128/mBio.01338-18 30131358 PMC6106089

[B64] Uruén C. Chopo-Escuin G. Tommassen J. Mainar-Jaime R. (2020). Biofilms as promoters of bacterial antibiotic resistance and tolerance. *Antibiotics* 10:3. 10.3390/antibiotics10010003 33374551 PMC7822488

[B65] Wilbanks K. Q. Mokrzan E. M. Kesler T. M. Kurbatfinski N. Goodman S. D. Bakaletz L. O. (2023). Nontypeable *Haemophilus influenzae* released from biofilm residence by monoclonal antibody directed against a biofilm matrix component display a vulnerable phenotype. *Sci. Rep.* 13:12959. 10.1038/s41598-023-40284-5 37563215 PMC10415356

[B66] Yang S. Li X. Cang W. Mu D. Ji S. An Y. (2023). Biofilm tolerance, resistance and infections increasing threat of public health. *Microb Cell* 10 233–247. 10.15698/mic2023.11.807 37933277 PMC10625689

[B67] Zemke A. C. D’Amico E. J. Snell E. C. Torres A. M. Kasturiarachi N. Bomberger J. M. (2020). Dispersal of epithelium-associated *Pseudomonas aeruginosa* biofilms. *mSphere* 5:e00630-20. 10.1128/mSphere.00630-20 32669459 PMC7364222

[B68] Zhao A. Sun J. Liu Y. (2023). Understanding bacterial biofilms: From definition to treatment strategies. *Front. Cell Infect. Microbiol.* 13:1137947. 10.3389/fcimb.2023.1137947 37091673 PMC10117668

