# Supplementary material for: Human PMNs exhibit greater engulfment, NETosis, and enhanced migration when incubated with nontypeable Haemophilus influenzae newly released from a biofilm
Source: Front Microbiol. 2025 Nov 27;16:1728903. doi: 10.3389/fmicb.2025.1728903 (PMC12695831; doi:10.3389/fmicb.2025.1728903)
Supplement: Supplementary file 1 [file Data_Sheet_1.pdf]

Supplementary Material

1. Supplementary Figures

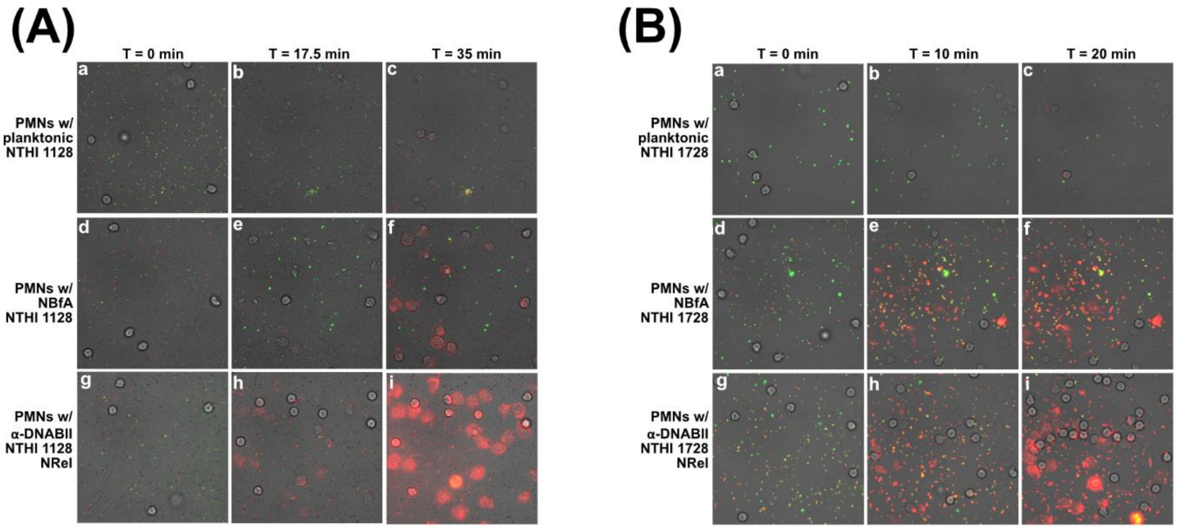

Supplementary Figure 1. Relative PMN activity in the presence of three distinct pHrodo™- and FM 1-43-labeled populations of NTHI 1128 **(A)** or NTHI 1728 **(B)**. By timelapse microscopy, there was evidence of PMN-mediated uptake and NETosis for all three tested populations of NTHI. Bacteria fluoresce green from FM 1-43 labeling which subsided once taken up by PMNs over the imaging period. Uptake of bacteria, NETosis, and red fluorescence were all visually greatest upon co-incubation of PMNs with α-DNABII NRel of both NTHI strains. Results are representative images based on three separate assays. Collectively, these data indicated greatest PMN uptake and NETosis activity with specifically α-DNABII NRel NTHI, regardless of strain assessed.
